# Partnership for International Development: Finland-Nigeria Conference on Climate, Food, Health and Entrepreneurship

**DOI:** 10.3390/ijerph19063375

**Published:** 2022-03-13

**Authors:** Dele Raheem, Oluwatoyin B. Oluwole, Leena Faven, Frank A. Orji, Mikko Junttila, Egidija Rainosalo, Sulaimon B. Kosoko, Adeola Olukosi, Seppo Vainio, Abiodun A. Olapade, Michael P. Okoh, Oyedele M. Oyeku

**Affiliations:** 1 Arctic Centre, University of Lapland, 96101 Rovaniemi, Finland; 2 Department of Food Technology, Federal Institute of Industrial Research Oshodi, Lagos 100261, Nigeria; orjifa@yahoo.com (F.A.O.); kosoko.sulaimon@fiiro.gov.ng (S.B.K.); deleoyeku88@gmail.com (O.M.O.); 3 RDI Chemistry and Bioeconomy, Centria University of Applied Sciences, 67100 Kokkola, Finland; leena.faven@centria.fi (L.F.); mikko.junttila@centria.fi (M.J.); egidija.rainosalo@centria.fi (E.R.); 4 Department of Medical Biochemistry, Eko University of Medical Sciences, Lagos 102004, Nigeria; yaolukosi@yahoo.co.uk; 5 Faculty of Biochemistry and Molecular Medicine, Kvantum Institute, University of Oulu, 90014 Oulu, Finland; seppo.vainio@oulu.fi; 6 Department of Food Technology, University of Ibadan, Ibadan 200284, Nigeria; aaolapade@gmail.com; 7 Department of Medical Biochemistry, College of Health Sciences, University of Abuja, Abuja 900105, Nigeria; michael.okoh@uniabuja.edu.ng

**Keywords:** innovation, sustainability, microbes, underutilized crops, processing, bioeconomy, circular solutions, food entrepreneurs, phytomedicine

## Abstract

A joint collaboration between the Arctic Centre of the University of Lapland, Finland and the Federal Institute of Industrial Research, Oshodi, Lagos, Nigeria was organised as a hybrid conference on several topics that are related to climate, food, health and entrepreneurship. The utilisation of natural resources in both regions is an important theme in meeting the sustainable development goals agenda. The topics discussed were multidisciplinary, they include Nigerian indigenous foods, bioeconomy, circular economy, nutrition, health, innovation and entrepreneurship under four themes (Climate, Food, Health and Entrepreneurship). There were dignitaries from Finland and Nigeria. The presenters are researchers from Nigerian universities (University of Ibadan, University of Abuja and Eko university, Lagos), Nigerian Federal Institute of Industrial research centre and from the Finnish side we have the university of Lapland, Rovaniemi, University of Oulu, Oulu and the Centria University of Applied Sciences, Kokkola. The topics discussed will serve as training materials for students and learners, the discussion focussed on research opportunities for institutions in both countries. The experts from both countries will continue to dialogue on the possibility of promoting common topics as research agenda in these important areas with the possibilities of creating more jobs.

## 1. Introduction

The urgency and complexity of global sustainability challenges, such as climate change, biodiversity loss, social injustice, and poverty, calls for new partnership in sustainability science [1]. The collaborative initiative between the Global North and the Global South is fostered through the University Partnership for International Development (UniPID) in Finland. UniPID is a network of Finnish universities to advance universities’ global responsibility and strengthen their response to global challenges. UniPID offers a variety of support services for the interdisciplinary studies, research and societal impact of universities that are related to global development.

FinCEAL develops Finnish Science, Technology and Innovation Cooperation between Europe, Africa, Latin America and the Caribbean through networking. FinCEAL BRIDGES contribute towards strengthening bi-regional cooperation, while expanding the thematic focus to the Agenda 2030 for Sustainable Development and the 17 Sustainable Development Goals, particularly Global Partnerships for Sustainable Development (Goal 17). In the efforts of the Nigerian government to add value to food crops which is regaining the desired attention in the country it will be important for the country to engage in value addition to the crops and innovate with new technology that can help to create more jobs among the youths in the country. The Federal Institute of Industrial Research, Oshodi, (FIIRO) Lagos, Nigeria being the foremost research institute in the country assists in accelerating the industrialization of the Nigerian economy through the utilization of the country’s raw materials and upgrading indigenous production technologies. The Arctic Centre at the University of Lapland is a national and international hub of information and centre of excellence that conducts multidisciplinary research in changes in the Arctic region.

The welcoming words were provided by the Director of the Arctic Centre, University of Lapland, the Ambassador for innovation, Finland and the Nigerian Honorary Consul to Finland. The presentations were based on self-reflection from the authors as a kind of autoethnography on the theme of this collaboration. There were two alumni from Finnish universities who are currently employed in two Nigerian institutions (University of Abuja and Eko university). A total of eleven topics were orally presented and discussed throughout the day, the papers included innovative research and review articles.

The theme of the conference ‘Climate, Food, Health, Entrepreneurship’ can help to trans-form a society when issues of environmental sustainability, poverty alleviation and social justice (partially codified in the Sustainable Development Goals or SDGs) are addressed through co-creation of knowledge.

## 2. Theoretical Considerations

The food and agriculture sectors must improve their sustainability performance and adapt to the impacts of climate change in ways that do not compromise global efforts to ensure food security for all [2]. These challenges are intimately and inextricably related, and need to be addressed simultaneously. As rightly observed in the Intergovernmental Panel on Climate Change (IPCC) Assessment Report, climate change leads to increase in temperature and precipitation variability, reducing the predictability of seasonal weather patterns, it also increases the frequency and intensity of severe weather events, such as floods, cyclones and hurricanes [3]. The IPCC emphasized that changes in climate and carbon dioxide concentrations will enhance the distribution and increase the competitiveness of invasive weeds. These will have consequences on food supply and food security. Agricultural diversification has an adaptive strategy to climate impacts, since diversity will help to increase resiliency of agricultural and natural systems, such as in resistance to increased pests or diseases; it can also provide diversified income portfolios when some crops may become sensitive to climate events [4,5].

A recent publication on low agricultural production in sub-Saharan Africa acknowledges that there is a wide range of edible crops that can be grown, but the hot and humid conditions make storage difficult, so crops that can be harvested daily or are easy to store are favoured, such as roots, tubers, legumes, plantains and cereals [6]. Therefore, the creation of knowledge that focus on post-harvesting operations that are climate smart will be appropriate. Nigeria as the largest oil producing country in Africa, have neglected agriculture on the discovery of oil but recently the agricultural sector is receiving a boost to diversify the country’s economy. The country is aiming to cut carbon emissions to zero by 2060 while in Finland this target is by 2035.

One Health is described as the concept that human, animal, plant, environmental and ecosystem health are linked. It has been recognized as an important strategy for examining and addressing complex global health issues [7]. As the Earth’s resources diminish and as human and livestock numbers continue to grow, the One Health policies are likely to become increasingly important to achieve the United Nation’s Sustainable Development goals. The importance of biodiversity within the One health approach was well recognized in preventing future crises of global pandemics and climate change. In October, 2020, more than 5000 people from 148 countries gathered online at the Global Landscapes Forum (GLF) Biodiversity Digital Conference: ‘One World-One Health’. The participants included indigenous leaders, policy experts, heads of global organizations, researchers from different disciplines, youth and other stakeholders to exchange knowledge and inform decision-makers about the crucial interdependencies of all life on Earth [8].

An understanding the various components that contributes to the total anthropogenic greenhouse gas (GHGs) emissions is a necessary precursor to the design and implementation of actionable and efficient mitigation measures for the system. The GHG emissions from food systems are far more than emissions from the land-based sector i.e., agriculture and food-relevant emissions from land use and land use change. Food needs to be farmed, harvested or caught, transported, processed, packaged, distributed and cooked, and the residuals disposed of [9]. The energy that are required for all these processes needs to be produced and made available at the right time and location.

The Joint Research Council of the European Commission developed an ‘Emissions Database for Global Atmospheric Research (EDGAR)’. EDGAR-FOOD was developed to aid the understanding of the activities that underlie energy demand and use, agriculture and land use change emissions that are associated with the production, distribution, consumption and disposal of food through the various stages and sectors of the composite global food system [10]. These data were complemented with data from the FAOSTAT database on GHG emissions from land use related to agriculture [11]. It represents the first database that consistently cover each stage of the food chain for all countries with yearly frequency for the period 1990–2015.

Both the EU and Africa will need to opt for a low-carbon, resource efficient and climate-resilient future in line with the Paris Agreement to achieve the Sustainable Development Goals. These facts are well recognised in the new Africa-EU Energy collaboration “Towards a comprehensive Strategy with Africa” [12]. Nigeria and other African countries are particularly vulnerable to climate change as it risks jeopardising the ongoing progress on sustainable development on the continent.

In this comprehensive strategy, innovation will be key to drive the green transition. Investments should be geared towards strengthening scientific capacities in Africa by providing access and local adaptation to technologies. Furthermore, trade can facilitate the adoption of innovative, sustainable business models that will play a leading role towards a climate-neutral future.

The co-production of knowledge on climate, food, health and entrepreneurship between Finnish and Nigerian partners in this conference can help to identify how this strategy can be better anchored to meet the sustainable development goals.

## 3. Materials and Methods

After initial contacts with Dr. Oluwole at FIIRO and Dr. Raheem’s virtual participation at the 45th Conference entitled “Food Security and Sustainability in a post COVID-19 era” and Annual General Meeting of the Nigerian Institute of Food Science and Technology (NIFST) which was held both on-line and physically from 11–15 October 2021 in Makurdi, Benue State, Nigeria resulting in this collaboration which is the first. The conference on ‘Climate, Food, Health and Entrepreneurship’ was held on 29 November 2021 in a hybrid mode as described below.

The hybrid event on 29 November 2021 was hosted physically with the aid of Jabra Speak 710 in Kieppi hall at the Arctic Centre of the University of Lapland, Rovaniemi and simultaneously in Akinrele hall at the Federal Institute of Industrial Research Oshodi, Lagos. There were thirty-six active participants that joined virtually and many others joined either physically or through Facebook streaming from Nigeria. There were eleven speakers from both countries (see Appendix A Table A1) who shared their thoughts for several hours throughout the day on several topics in both physical and virtual modes. The physical and virtual event on zoom meeting considered the geographical time difference between the two countries. The event was also promoted on Twitter with #AC_FIIRO to target audience that includes local research groups, farmers, food processors and other stakeholders within the food system in both countries and globally. The zoom meeting also featured breakout rooms where participants: (i) brainstormed on the concrete steps towards linking Universities and Industries collaboration on the food system with health implications (ii) the challenges and possible solutions for promoting traditional indigenous foods to communities in Nigeria. At the plenary, the breakout groups reported their findings. The whole event was recorded in cloud for future training and development opportunities by participating partners. All the topics discussed at the event are presented in the next section.

## 4. Results and Discussion

In this section, some authors have reported their topic in a research article format as Introduction, Materials and Methods, Results and Discussion, Conclusion while others have reported their topics as extended abstracts by highlighting the most relevant points of their talk. A list of the titles presented in the Results and Discussion section, Speakers and their backgrounds are presented as Appendix A Table A1. 

### 4.1. Processing and Utilisation of Lesser Known Crops as a Panacea for Food and Nutrition Security (Abiodun A. Olapade)

Food is a complex mixture of organic and inorganic chemical compounds which the body requires to grow and maintain itself in healthy conditions. Food security implies access to food of the right quality and quantity by every child and adult. Food security for a household means access by all members at all times to enough food for an active and healthy life. Eating good food is not necessarily costly but it requires choosing rightly among the available crops. There are hundreds of little-known indigenous crops that can contribute to food and nutrition security of developing countries including Nigeria. These little-known indigenous crops have played vital roles in nutrition of the people but they are underutilised. These can be attributed to many reasons including drudgery and time wasting of their processing into foods.

These lesser known and underutilised crops are abound in Nigeria and other African countries [13]. Examples are tubers such as water yam, bitter yam and cocoyam; legumes such as Bambara groundnut, African yam bean, Ackee apple seed, Pigeon pea, Tiger nut and groundnut; Cereals such as Acha, Finger millet, Amaranths seed etc.

Food processing is the transformation of agricultural produce into intermediate food materials or finished food products through the use of appropriate techniques. Food processing comprises various unit operations that help to transform the raw material into a predictable product. One of such food processing techniques is extrusion cooking technology. Extrusion cooking is capable of producing unique food products from array of raw materials. The process is capable of:

− Form and shape food of different types
− Modify chemical and physical properties of foods
− Destroy microorganisms in foods
− Inactivate enzymes
− Destruction of heat liable anti-nutrients
− Degradation of microbial toxins in foods

With the use of extrusion cooking technique, it is possible to formulate nutritious and acceptable food products from the array of the lesser known and underutilised crops mentioned above. Thus, problems of food and nutrition insecurity, postharvest losses of the crops and empowerment of smallholder farmers as well as women especially in rural areas will be tackled headlong.

### 4.2. Microbial Food Safety Concerns: Control Measures from HACCP Plans (Frank A. Orji)

The review paper through Google search, PubMed, and other relevant search engines x-rayed the concept of food safety and the central roles of HACCP in the global control of food contamination and intoxication. Most countries have documented significant increases in the incidence of diseases caused by microorganisms in food over the past few decades. Culture-independent techniques (metagenomics) have helped to change the way to study food microbial ecology, leading to consider microbial populations as consortia. The use of HACCP measures has been suggested as a major instrument for the control of these food borne outbreaks.

#### 4.2.1. Introduction

The contamination of food by microbial agents is a worldwide public health concern. Most countries have documented significant increases in the incidence of diseases caused by microorganisms in food over the past few decades [14]. Microbial hazards in food include bacteria such as Salmonella, viruses such as Norovirus, parasites such as trematodes as well as prions. Diarrheal diseases are the most common illnesses resulting from the consumption of contaminated food, causing 550 million people to fall ill and leading to 230,000 deaths every year. In addition, diarrheal diseases may cause malnutrition and stunting, adding to the amount of Disability-Adjusted Life Years (DALYs) resulting from the consumption of contaminated food [15].

#### 4.2.2. Materials and Methods

This is a review paper, where search engines like google, altavista.com, mama.com and Elsevier were used. The key words used in the search were Food infection, contamination, intoxication, HACCP, and metagenomics.

#### 4.2.3. Results and Discussion

A look at emerging and re-emerging food borne microbial pathogens and diseases: Emerging food borne diseases are defined as diseases that are food transmitted, whose incidence in humans has increased in the past two decades. They are characterized by reduced incidence reduced mortality rate in human race due scientific recognition [16]. Examples of the emerging food borne diseases include but not limited to *E. Coli* [17,18]. Diarrhea, Salmonellosis, Campylobacteriosis, Cryptosporidiosis, Shigellosis, Yersiniois etc. Re-emerging food borne microbial diseases are food transmitted microbial diseases that re-appear after a significant decline in incidence thereafter a long period of science –based record of decline in incidence [16].

#### 4.2.4. Conclusions

Microbial food safety has been a major concern in the globe as a result of re-occurring out breaks of diseases of microbial origin. The laboratory identification of food borne pathogens through surveillance has been laborious and consumes more time until the introduction of metagenomics approach. This culture independent approach (metagenomics) consumes less time and is very effective in detecting microbial pathogens in food samples. The use of hazard analysis critical control points (HACCP) measures has been suggested as a major instrument for the control of these food borne outbreaks.

### 4.3. Food Processing: Implication on Nutrition and Health (Sulaimon B. Kosoko)

Food processing has been an age long practice that contributes immensely to both food and nutrition security of the populace. Over the years, the objective and purpose of food processing has shifted from improving the safety, nutritional value or extending the shelf life of food alone to increasing the convenience and palatability of food at various homes especially in industrialized high-income countries. However, the increase in the desire for fast, convenient and pre-processed food by consumers has led to the transformation of the food industries; food industries now intensify the grade of their processing by developing the so-called “Ready-To-Heat” or “Ready-To-Eat” food or dishes. Today’s high-intensity industrial processed foods often exhibit higher concentrations of added sugar, salt, energy but lower micronutrient density which adversely affect the health of consumers; resulting in diseases like obesity, chronic and degenerative noncommunicable diseases. Nowadays, consumers are more health conscious and perceive food as not only a source of energy but also as necessary tools for maintaining good health. They do not want fully or highly processed food rather preferring minimally processed food; hence challenging food processing industries on finding ways to decrease the risks of food processing induced diet-related noncommunicable diseases while making progress on food and nutrition safety. Also, due to the health awareness of consumers, natural or minimally-processed foods have now occupied an important place in the today’s consumer market, and many industries (especially in the developed world) have begun to promote the utilization of physical non-thermal food processing methods (high-pressure processing (HPP), ultra-high voltage (UHV), pulsed electric field (PEF), cold atmospheric plasma (CAP), microwave, ohmic heating (OH), ultrasound etc.) as a more sustainable alternative methods of food processing that would preserved the essential nutrients in commercial food products. Conclusively, the continuous rise, adoption and utilization of non-thermal physical processing technology in food industry would perfect the act of “Nutrition-Sensitive Food Processing” and enable food industries to find the balance between maximizing the nutrient output of food processing systems and production inputs while minimizing costs.

### 4.4. Value Addition to Indigenous Foods for Food and Nutritional Health Security and Job Creation (Oluwatoyin B. Oluwole)

#### 4.4.1. Introduction

Value addition to agricultural produce can be defined as a process in which a high price is realized for the same volume of a primary product by means of processing, upgrading the quality, packaging and other such methods. Agricultural produce such as fruits and vegetables, roots and tubers, cereals and legumes have been reported to be highly perishable with short postharvest life and high moisture content (65–70%) at harvest [19] with resultant huge annual post-harvest losses. Nigeria produces at least 2,722,000 metric tonnes of plantain annually [20], at least 2 million metric tonnes of onion as reported by [21], 850 metric tonnes of mango was produced in Nigeria according to [22], 3.46 million tons per year for sweet potato annual production in Nigeria [23], 0.1 million tonnes of annual bambara groundnut production as reported by [24] but the country is still food and nutritional health insecure with attendant high prices of foods at off season period as well as limited job opportunities for stakeholders. This paper highlights the value addition prospects especially in relation to indigenous selected food crops in Nigeria that are capable of enhancing food and nutrition security, minimize hunger, alleviate poverty in relation to the United Nations Sustainable Development goals by year 2030 and create more job opportunities in Nigeria.

#### 4.4.2. Methodology

Extensive literature review on the selected crops in the area of current value addition practices, the nutritional health benefit potential from each crop and their associated waste materials upon processing were studied. Current job opportunities based on existing postharvest practices for each selected crop was also established.

#### 4.4.3. Results and Discussion

There is an urgent need to standardize the primary processing technologies, storage technologies, packaging of each crop and their intermediate processed forms where applicable with direct connecting links among farmers, processors and consumers as much as possible. The major job opportunities are in the area of on farm processing, packaging and storage, marketing and processing into finished products. Novel product development in the area of utilization of wastes such as plantain peel [25], mango peel [26] and sweet potato leaves [27] could also assist in promoting more job opportunities especially in the area of functional and nutraceutical foods due to their high dietary fiber and antioxidant content.

#### 4.4.4. Conclusions

Standardized and sustainable value addition to the selected agricultural produce are packed with micro and macro nutrients and bioactive compounds with established antioxidant activities could promote food and nutrition security, nutritional health and job creation opportunities for the populace.

### 4.5. Phytomedicine in Disease Management: A Case of In-Vivo and In-Silico Analysis (Michael P. Okoh)

Nature provides rich sources of structurally diverse phytochemicals with medicinal relevance and biological activities. Sodium arsenite has been recognized as a worldwide health concern due to its teratogenicity effects on animals. Natural plants are considered as a possible protective agent against arsenic induced toxicity. We investigated the protective effects of Delonix regia leave extracts on sodium arsenite induced hepatotoxicity in rat. Animals were randomly divided into six groups of five per group. Group A (control) received distilled water for fourteen (14) days. Group B received 2.5 mg/kg body weight Sodium Arsenite, group C received 100 mg/kg of leave extract and 2.5 mg/kg of sodium arsenite, Group D received 400 mg/kg of leave extract and 2.5 mg/kg of sodium arsenite, Group E received 100 mg/kg of leave extract, Group F received 400 mg/kg of leave extract only. On the fourteen day all the animals were sacrificed. Biochemical parameters such as plasma Alanine amino transferase (ALT), Aspartate amino transferase (AST) Alkaline phosphate (ALP) and gamma glutamyl transferase (GGT) total protein (TP) were evaluated. Sodium arsenite in rat triggers significant increase in ALT, AST, ALP, TP, and GGT (*p* < 0.05 level). Upon treatments with Delonix regia leave extract, it decreases the concentration of GGT, AST and ALP compared to the negative control. Group treated with the extract alone showed no adverse effects on the liver parameters. Results from this study suggest that, the administration of Delonix regia leave extract confer some protective effects on the liver [28].

Similarly, more recently using phyto-compound, we try to understand the complex life cycle of mosquito malaria transmission and their involvement in cerebral malaria via synaptic binding. To this effect, we carried out a simulation using molecular dynamics, binding free energy estimations and relate this with phytochemical properties of the plant (Neem), and compared it with the binding affinity of Artesunate and Azadirachitin to Gephyrin E. We, were able to show that the bioactive components of the Neem plant properly harnessed, may be very effective for the management of malaria disease phenotype [29].

Keywords: Malaria, Molecular dynamics, Gepherin, Hepatotoxicity; Delonix regia; Leaf extract; Phytomedicine.

### 4.6. Nigerian Indigenous Lipid Foods and Cardiovascular Health (Adeola Olukosi)

In this presentation on cardiovascular diseases (CVD), the basic facts on fat as a high energy yielding reservoir that accounts for 35–45 % of caloric intake was highlighted-1 gm of fat being equivalent to 9 Kcal. The daily requirement of fat is not known with certainty, infant diet is slightly over 50% of total energy intake. Fats are a vehicle for fat soluble vitamin (A, D, E and K), precursors for essential fatty acids such as linoleic acids. They are also necessary for the synthesis of cholesterol, prostaglandins, testosterone and other lipid-containing hormones and compounds. There are lots of conflicting information in the literature as to the acceptable or desirable components of health and unhealthy components of fats and oils and their attending implications. The weight of these evidences in arriving at a resolution concerning what ought to be acceptable needs to be well defined by standards that are peculiar to Nigeria. Dyslipidemia is a well-documented risk factor for cardiovascular disease. The glycemic indexes of foods and those with high glycemic load have been considered unhealthy such as amala, gari, ripe plantain, alcoholic drinks, soft drinks, white bread, doughnut, pancakes, pizza, white short grain rice, millet, tuwo sikafa and masara. Over 80% of the global burden of CVD occur in low- and middle-income countries. Cardiovascular diseases accounts for nearly half (48%) of deaths due to non-communicable diseases (NCD). In Nigeria, NCDs account for about a quarter of all deaths annually while CVDs are responsible for 7%; being the highest cause of deaths due to NCDs [30,31]. Coronary heart disease deaths in Nigeria reached 53,836 or 2.82% of total deaths, and are responsible for the greatest proportion of the total mortality from non-communicable diseases [32].

Nigeria as a multicultural society with different traditional soups that are indigenous to the different ethnic and cultural society. Most Nigerian soups have meat, fish, palm oil, little or no vegetables, crayfish, seasonings and water as their constituents.

A typical Nigerian meal is heavy with starchy items (cassava flour, rice, cocoyam, potatoes, yam or plantain), very small amount of protein and generous on fat such as palm oil or other vegetable oil in soups with or without vegetables Miyan-kuka with semovita (415.9 mg per 100 g) contain high amounts of free fatty acid and cholesterol. Other examples are: Stewed beans with fried plantain had the high total lipid (86.5 g per 100 g) content, yam with fried eggs has a high triacylglycerol (122.5 mg per 100 g) contents, local snacks (akara) have higher amounts of cholesterol, Ofada stew is high also in cholesterol and triglyceride, Ogbono soup without vegetables high in cholesterol and triglyceride, plain egusi stew more detrimental than those mixed with vegetables, Jollof rice [33,34].

In addition, many brands of vegetable and animal oils are manufactured and or marketed in Nigeria without appropriate labelling of their constituents and origin (plant or animal source). Raw red palm oil is a rich source of phytonutrients, carotenoids, tocopherols, tocotrienols, sterol, phospholipids and polyphenols. Palm oil also contains 40% oleic acid which is monounsaturated and a major constituent of olive oil. The Nigerian Heart Foundation recommends: DASH (Dietary Action to Stop Hypertension) diet that are rich in vegetables, fruits and wholegrains and foods low in oils and fats are recommended. Healthy protein sources (especially fish and seafood), legumes (such as beans and lentils), nuts and seeds. Smaller amounts of eggs and lean poultry, lean red meat limited to 1–3 times a week can also be included in a heart healthy diet [35].

There is a gap of scientific knowledge that needs to be filled on lipids and cardiovascular health in the Nigerian population. The consensus on all the points arrived at by a summit on cardiovascular health in the Nigerian population summarised most of the pertinent issues [35]. The high amounts of total lipid are associated with higher social economic factors in the privileged but not necessarily traditional Nigerian dishes and is responsible with severe health implications and high incidences of diet-related chronic diseases.

Ultimately, awareness creation is important and the Federal Government of Nigeria needs to continue to play a coordinating role in convening stakeholders meeting consisting of philanthropic foundations, nongovernmental organizations, research institutions, private companies, industries and international institutions to address NCDs periodically. This will help to develop a strategic and sustainable plan for collective actions on NCDs in Nigeria.

### 4.7. Innovation and Technology Options for Micro and Small-Scale Investment: Preference for Food Entrepreneurship (Oyedele M. Oyeku)

#### 4.7.1. Abstract

The advent of COVID-19 has changed the way we do things including working from home and digitalization of business, meeting and so on. Today, we are deploying innovation to every aspect of human endeavor. At FIIRO, we are developing innovative programmes to ensure that workers working from home are working effectively. One of such innovative programmes is the Neighborhood Technology Extension Services Program where staffs are required to identify organizations in and around the area they are living to make presentations on innovations/technologies available at FIIRO for micro and small scale enterprises development. The power point presentation designed for this purpose consists of 100 innovations/technologies developed at FIIRO which are low hanging fruits for micro and small enterprises (MSEs) development. Forty of these innovations/technologies are food-based while 60 are non-food innovations/technologies. At the end of each presentation, questionnaires were administered to participants to indicate their preference for innovations/technologies for entrepreneurship. Questionnaires administration and retrieval took place between March, 2021 and October, 2021 in thirty locations. Out of 2205 administered questionnaires, 1860 were retrieved and analyzed. The result indicated that 67.8% of the total respondents have preference for food-based innovations/technologies for the purpose of investment while 32.2% have preference for non-food innovations/technologies. Gender played significant role amongst respondents having preference for food-based innovations/technologies with 61.2% females having preference for food-based innovations/technologies compared to 38.8% for males.

Keywords: Innovations, Technologies, Food Entrepreneurship, Micro and Medium Enterprises.

#### 4.7.2. Introduction

The advent of COVID-19 and its declaration as pandemic by the World Health Organization (WHO) has affected all areas of human endeavors including economy, policies, social behavior and mentalities of citizens [36,37,38,39].

The Presidential Task Force on COVID-19 was set up in Nigeria with the objective to mitigate the impacts of COVID-19 on all aspects of human endeavors. As a response to curtain the spread of the virus, the Task force directed that workers especially those on Grade Level 13 and below should work from home beginning from December, 2020 but a new circular was recently issued by the Head of Service of the Federation that all categories of government workers should resume duty effective from Wednesday, 1st December, 2021. The Institute developed the Neighborhood Technology Extension Services Program to keep staff working effectively from home by identifying some organizations in and around their neighborhoods to make presentations on FIIRO’s developed innovations/technologies that are available as low hanging fruits for investment opportunities towards micro and small enterprises development with the objective to increase the adoption rate of the Institute’s technologies, promote entrepreneurship as well as enhance socio-economic development of Nigeria.

This paper presents the results of the analysis of the “Questionnaire on Technology of Interest” which was administered to participants during each of the presentations and its implications for food entrepreneurship development in Nigeria.

#### 4.7.3. Methodology

The Neighborhood Technology Extension Services Program works on the assumption that staffs are well familiar with the neighborhood where they reside and they can identify relevant organizations within their neighborhoods to make presentations on “Innovations/Technologies Available at FIIRO as Investment Opportunities for Micro and Small Enterprises Development”. A power point presentation consisting of 100 innovations/technologies (40 food-based and 60 non food-based innovations/technologies) was designed for this purpose. Data collection was done using questionnaires administered between the month of March and October, 2021. Section A of the questionnaire captured the biodata of the respondents while Section B captured the technology interest of the respondents. The retrieved questionnaires were analyzed using simple percentage method.

#### 4.7.4. Results and Discussion

Male (64.8%) dominated the gender that attended the various presentations in the 30 locations under study while large percentages of students (20.1%), public servants (27.4%) and business owners or people working in private sector (40.3%) participated probably as a result of trying to find every possible means to mitigate the negative effects of the pandemic. Also, about 59% of the attendees have tertiary educational qualifications which could imply that they really know what they wanted and would like to pursue their entrepreneurship dream being the major goal of the presentations. A total of 67.8% of the total respondents indicated preference for enterprise development around food-based innovations/technologies. This observation or trend could be linked to increasing development of food entrepreneurship most especially at this period of pandemic. Food business appears to be the business of the pandemic era; everyone must eat even when you are working from home. Food appears to have no alternative. It was also observed that out of the respondents that indicated preference for food-based innovations/technologies, 61.2% of them are females.

#### 4.7.5. Conclusions

The study concludes that there is an increasing preference for investment in food entrepreneurship especially at this pandemic period. It also concludes that more females than males are interested in food entrepreneurship. There is a need therefore, to develop appropriate curriculum for food entrepreneurship to impact the necessary skills on would be food entrepreneurs.

### 4.8. Health Promoting Products from Non-Wood Forest Products. Opportunities for Growth in the Nordic Bio-Economy (Leena Favén)

Leena Favén gave a short introduction on the current status of Finnish bio-economy. The Ministry of Labour and Economy estimated the total turnover of natural products sector in 2019 was 500 million euros while the Natural Resources Institute (Luke) estimated value addition from bioeconomy to be 26 billion euros in 2020. The forecast for the global demand for health promoting products from non-wood forest products (NWFP) such as essential oils, nutraceuticals and antioxidants is estimated to be over 350 billion US dollars in 2021 [40]. In Finland, bio-based raw materials such as cultivated and wild plants as well as forest and side streams from the food industry have not been utilized to their full extent and there is a great opportunity to refine high value-added health promoting products that can be utilized as functional food, food supplements, cosmetics and pharmaceutical products from these high quality Arctic raw materials that are grown in clean environment.

Leena Favén also introduced the White paper by The European Forest Institute and the Food and Agriculture Organization of the United Nations [41] ”Non-wood forest products for people, nature and the green economy. Recommendations for policy priorities in Europe”. According to the White paper, the market value of non-wood forest products (NWFP) in Europe: is 23 billion euros annually. Current risks and threats regarding the development of NWFP sector are e.g., climate and land use changes, uncontrolled harvesting, illegal trade and competition with fossil based or non-renewable alternatives. The following recommendations are given in the White paper in order to enhance sustainable utilization of NWFP raw materials: secure conservation and sustainable supply of non-wood forest products, build competitive and sustainable value chains and improve transparency.

The extraction and characterization of valuable compounds from bio-based raw materials is one of the research and development interests at the Chemistry & Bioeconomy team of Centria University of Applied Sciences which enhance the development of industrial refining of high value added bio-based products. Concentrations of polyphenols and antioxidant capacities have been analysed in order to characterize potential health promoting properties and premium quality of various bio-based raw materials of Nordic berries, leaves and herbs.

Looking ahead beyond the conference, some project ideas that were presented for future learning and collaboration opportunities include the following:

− climate change challenges and how to meet global challenges on the availability of sustainable plant-based raw materials in order to provide food for growing and ageing population
− conduct comparative studies on plant-based health promoting ingredients that are utilized in different parts of the world and countries e.g., Nigeria and Finland
− how to work towards the harmonization of global standardization of methods that will ensure quality and authenticity.

### 4.9. Adopting Circular Solutions for Small Breweries (Egidija Rainosalo, Mikko Junttila)

Many cultures across the globe brew alcohol-containing drinks from the past to date. It will be important to ensure that less waste is generated during the brewing process. Therefore, circular solution that has a potential for Finnish breweries might also be well executed in other countries.

The main ingredients in brewing as inputs are malt, water, hops and yeast. Additionally, breweries are both energy and water-intensive industries [42]. A cleaner production is continuously advocated in order to reduce consumption and emissions from production process, products and services during production in the brewery industry. Energy is needed for heating and boiling liquids, cooling requires a substantial amount of cooling water. Since it is only the liquid product is of interest, the main by-products of the process are brewers spent grain (BSG), spent hops/trub and spent yeast. BSG is the major by-product, representing around 85% of the total by-products generated. 100 hl of beer would produce 2 tons of wet spent grain which usually contains 10–20 % of dry matter. In addition, the fermentation process produces CO2 which in most cases is emitted into the air.

The authors considered the current status on the use of these by-products, challenges and future outlook. In Finland, the number of small breweries is on the rise, small breweries can improve their economy and still have a positive environmental impact by implementing the principles of the circular economy. The main challenges are associated with setting up a value chain which is economically feasible and how to find high-value products. Many Finnish breweries are located in rural remote areas and they have small outputs, i.e., producing less than 1000 hl of beer per year. The most important passion for entrepreneurs engaged in these breweries is quality and taste, less emphasis are put on the efficiency of the brewing process and advanced handling of side and waste streams. In order to meet the challenges and ensure circularity, value chain organisers and orchestrators or match-makers are needed to assist entrepreneurs in finding stakeholders in the ecosystem for upgrading the by-product to higher-value products.

Finnish beer is normally produced from barley. Barley crop is rich in proteins and dry BSG contains about 15–30% of crude protein. This fact is well recognised by farmers and if cattle farms are close to breweries, the by-product is picked up by them and they can be used as feed for animals. Such collection practices aid a potential reduction of biowaste handling costs, such as transportation and gate fee of biowaste acceptor. Other minor routes for BSG include biogas production, fertilizer and composting. Spent yeast from breweries can be sold as source of proteins and B-vitamins.

Therefore, higher value products that can overpower pre-treatment and transport costs would inspire entrepreneurs to create value chain for better utilisation of this by-product. Collaboration will help to scale up the by-products from small breweries and reduce costs associated with remote locations.

Centria University of Applied Sciences is assisting the brewing industry in the estimation of the technological routes and identification of potential partners for collaboration in BSG, as well as other by-products, utilisation. The available technology includes:

− preservation of BSG since it is highly susceptible to microbial growth and spoilage,
− chemical and enzyme-assisted separation of proteins from BSG,
− the identification of other by-product sources to increase the scale of the technologies that are being developed.

We expect that the work will help to inspire industries in creating novel circular economy, inspire business cases and improve the resource efficiency of bio-based materials. The work is financed by European Union’s Interreg NPA Programme as part of SYMBIOMA project.

### 4.10. Towards Evolutionary Conserved Biological Nanofunctional Superfood and Therapeutics (Seppo Vainio)

Nature provides the fundamental foundation of our lives by nourishing us. We also obtain wealth from raw materials, build our living conditions and many other societal aspects of our lives. During development of human culture one essential element has been to learn to produce food products in larger quantities. Much of the development of the food production and processing technologies have been developed to increase production yields, longer transport and storage duration. In view of this, food products have been only seen from the point of view of calories, vitamins, fibers, proteins, sugars and lipids that are obtained from the food we eat. However, science has identified that nature including the food we eat, and the microbiome of our gut are organized also at the nano- and microlevel. What this means is that most of not all of the live cells secrete small, that is nano- and micro size extracellular vesicles (EVs).

The EVs provide a new biological information transfer system in nature since EVs carry a wealth of analytes, including many RNA, DNA, protein, lipid, sugar and metabolite species. The EV information transfer system seems universal and they are thus present in the food products. The EVs will have an impact on the way we prepare food. Given this and our current knowledge, I speculate that many of the current food products prepared and sold do not respect the delicate nano biological EV elements. Thus, I speculate that diseases, especially those of the bowel/intestine ones may be promoted by the mass production technologies of foods that destroy/deregulate the nano- and microstructure EVs. We can test the hypothesis with our sophisticated nano- and micro biochemical and cell biology technologies. We are able to purify the EVs, identify in part their content molecular and test their biological cargo capacities in cultured cell, organoids and in the defined preclinical in vivo models. My team at the University of Oulu master these approaches for the EV studies. The discovery of the secreted nano- and micro vesicle EVs, or exosomes is still a rather new field [43]. Based on what we know of them they have opened up a wealth of existing research but also application opportunities. There are several companies that offer services and reagents for the EV field. The expectations for the nanobiology and nanomedicine field is illustrated well by the 1 billion € Roche company medical investments to milk EVs only exposed orally to the body to transfer drugs to human cells across the gut [44].

### 4.11. Transforming the Food System through Digital Solutions: Perspectives from Finland (Dele Raheem)

The food system is a digital lagger when compared to other systems such as the health sector. However, many start-ups are coming up to fill this gap in the food sector. It has been estimated that the industrial food system requires 10–15 energy calories to produce one calorie of food, accounting for 22% of greenhouse gas (GHG) emissions [45]. The food system is responsible for one-third global anthropogenic GHG emissions and the food sector will need specific sectorial energy efficiency and decarbonisation policies towards mitigation [46]. The recent 2021 UN Food System Summit Scientific Group recognized that ‘Food’ is central to People, Planet and the sustainable development goals (SDGs). In order to reach this goal, an improvement in the performance of our food system will be very crucial to reaching the Sustainable Development Goals. How we process, consume and dispose food waste and packages need to be more responsible and sustainable. Currently, the green economy requires the manufacturing industry to gradually transition from the era of mass production into the era of smart production, where physical production is merging with the opportunities created by digitalisation into cyber-physical systems. For example, from the Finnish Lapland perspective, the concept of smart specialisation is designed to ensure that the Arctic natural environment is turned into an opportunity in the form of natural-resource utilisation, from which new and innovative business areas and networks are emerging alongside and within the traditional industries [47]. Digital solutions within the food system can help to minimize environmental impacts and ultimately improve sustainability. In meeting the demand of local consumers, distributed and localized manufacturing will help to reduce food miles and promote food sovereignty in local food systems. The adoption of digitalisation at different stages of the food value chain will open up market accessibility for locally produced food products in local communities. Digital solutions will have major impacts on the local food system as it creates better opportunities to innovate and promote local foods with high quality before they end up in the market, this will also demand constant supply and availability of these foods. Availability will be best guaranteed with support to growers, processors and other stakeholders that are involved in the food value chain. The presentation also focused on some of the innovative aspects from the project on ‘Digital architecture as a roadmap for local food business operators in Finnish Lapland’.

Food Economy 4.0 as an ecosystem connects both traditional and emerging actors with end users in new ways. In Finland, the know-how of biomaterials, modular processes, robotics and digital technologies will create new international business opportunities, but they can also improve the competitiveness of domestic food [48]. When data are collated on what is happening at each stage of processing, it will provide useful information that will help artisan food producers at local levels. With accurate data to inform, the value chain of local products will create opportunities that can improve on these processes at different stages. It will also be easier to share best practices and monitor food safety. The incorporation of big data with the latest technology will help to drive, map and integrate data from across the whole food supply chain, e.g. from weather and remote sensing in agriculture, to tracing the origin of raw ingredients, the nutritional quality of foods, tracking how food has been produced or handled. These innovative breakthroughs are making their ways to the future food system with smart labels on food packages that can be scanned to reveal a host of information about a food product, that allow consumers to differentiate between products based on health and sustainability parameters [49].

## 5. Implications and Future Perspectives from the Collaboration on Development

Some future perspectives on the above topics are summarized as follows:

4.1: The important role of lesser known crops to support food and nutrition security, will help smallholder farmers to ensure biodiversity and cultivate more of such crops in the future.
4.2: HACCP is important to ensure food safety especially when cottage industries are considered. The means of ensuring these points are strictly adhered to can be enhanced digitally.
4.3: The need to pay particular attention to the extent of processing and its effects on essential nutrients, natural foods with less preservatives and chemical additives.
4.4: The revival of indigenous foods that are acceptable culturally when developed will be useful, need to improve the quality standards, ensure safety. A wide range of the suggested crops and their nutritional benefits can be better highlighted with innovative research. Agro-processing at small scale with affordable and appropriate technology with government policy that supports such initiatives.
4.5: The advancement of medicinal plants as therapeutic drugs from the laboratory to market need more research support.
4.6: ‘Food as medicine’, will require the need for better nutrition education across the Nigerian populace that will help to prevent cardiovascular diseases.
4.7: The opportunity to adopt latest tech. and innovation to support food entrepreneur amongst the teeming young Nigerian population.
4.8: Efficient drying technology techniques will be useful to limit damage to freshly harvested crops and natural plants with health-giving properties.
4.9: Small, artisan micro-breweries will help to reduce food miles, ensure circularity and help with climate mitigation.
4.10: Future research that involve exosomes in cells can be therapeutic especially when functional foods are considered.
4.11: Digital tools can serve as essential tools within the food system for efficiency, labelling to communicate with consumers,

Overall, the innovative ideas presented at the event with support from policy makers will help to advance development. As recently observed, that countries that align their policies on open innovation, with environments dedicated to research and development stand out more than others [50]. The break out and plenary sessions devoted time to the role of the students’ industrial work experience scheme (SIWES) at tertiary institution in Nigeria as a development that need to be improved upon such that students can have the opportunity to investigate traditional and indigenous food crops.

It was also highlighted that the possibility of forming cooperatives along the food value chains where there is competitive advantage at regional level will be worthwhile investigating. The opportunity can be extended to the West African region, e.g., Nigeria and Ghana can prioritize a food crop to jointly develop and increase the supply base and its processing in their different communities. The packaging and quality standards can be well harmonized with inputs from Finland.

There is a need to cultivate more indigenous food crops and to diversify into new products by using processing and preservation techniques such as (extrusion technology described in 4.1) to ensure that more people have access to good quality, nutritious foods. It is therefore essential that agro-allied industries in Nigeria receive encouragement to improve the application of technology for processing raw food crops, increasing their shelf life, improving nutritional vaues and packaging, and maintaining high quality standards [51]. 

It is worthwhile mentioning the positive example of Nigerian Zobo drink from Hibiscus sabdariffa L. This drink has been widely used traditionally as a food, in herbal drinks, in hot and cold beverages, as a flavouring agent in the food industry and as a herbal medicine in local medicines in many countries. In India, Africa and Mexico, infusions of the leaves or calyces are traditionally used for their diuretic, cholerectic, febrifugal and hypotensive effects. There are research papers that confirm that it has the ability to decrease the viscosity of the blood and stimulates intestinal peristalsis [52]. It is also recommended as a hypotensive in Senegal [53]. In India, a decoction from the seeds is used to relieve pain in urination and indigestion. In Brazil, the roots are believed to have stomachic and emollient properties. In Chinese folk medicine, it is used to treat liver disorders and high blood pressure [53]. In Egypt, preparations from the calyces have been used to treat cardiac and nerve diseases and to increase the production of urine i.e., diuresis. In Egypt and Sudan, an infusion of “Karkade” calyces is also used to help lower body temperature [54]. In Guatemala, it is used for treating drunkenness [53]. In North Africa, calyces preparations are used to treat sore throats and coughs, as well as genital problems, while the emollient leaf pulp is used for treating external wounds and abscesses [55].

The planetary boundary framework as described by Steffen and co-workers contributes to this paradigm by providing a science-based analysis of the risk that human perturbations will destabilize the Earth System at the planetary scale [56]. By addressing sustainability issues in the management of global resources and co-creating knowledge in the theme of this conference can help to shape development in our changing planet. The One Health framework described under Section 2 “Theoretical considerations” can be further promoted as a multi-dimensional matrix that can provides users with a tool to research, analyze, and address complex health threats in a concise, systematic, and comprehensive way. In this context, digital tools have an important role as we can think beyond traditional boundaries. Digital solutions such as precision agriculture will act as drivers of agricultural growth and transformation while also helping to raise the demand for innovations. When digitalisation is supported with the right policies at governmental level, food sovereignty will help be strengthened. One of the most important solutions is using information and communication technology (ICT) to transform the food system in the Global South as in Nigeria. ICT will help to improve agricultural technologies, increase food production, markets, banking, and financial services that are related to the food system. Training of the youth as ‘agripreneurs’ will equip them with skills and the use of ICT improves their creativity towards problem-solving for the betterment of the agri-food value chains [57].

However, there are challenges that may be obstacles in achieving progress in this partnership. First, socio-cultural barriers that are related to food options. This was rightly captured in the work of Macdiarmid and colleagues, they asserted that while diets have changed in the past, they are deeply culturally embedded and behaviour change is extremely difficult to effect, even when health benefits are well known [58,59]. The demography of the two countries is also an issue. According to Worldometer (2022), Nigeria has a population of 214 million with an average age of 18.1 years while Finland has a population of 5. 5 million with an average age of 43. 1years [60]. The level of infrastructural development has an influence on other socio-economic activities such as manufacturing and value to foods, access to efficient and smart energy. Dedication to research on value addition to Nigerian indigenous crops will need better support from research fundings. The availability of capital to engage businesses particularly small and medium enterprises that are economic drivers can be improved with the right policies.

## 6. Conclusions

The various topics (Appendix A Table A1) that were discussed at the event are relevant to promoting health, ensuring a sustainable use of natural resources, mitigating the impacts of climate change and seek ways that will help to provide more jobs in the agro-allied sector. Clearly, there is a need to further promote university-industrial linkages through the students work experience schemes especially in Nigeria. Such efforts can be championed by the Nigerian Federal Institute of Industrial Research Oshodi (FIIRO). The researchers in both countries look forward to developing proposals that can be of mutual benefits in the future. The traditional and indigenous food crops in Nigeria need to be promoted, the traditional knowledge behind their processing can be revisited with the aim of moving from cottage levels to industrial processing. In addition, the researchers emphasized the need to diversify food crops in Nigeria through more efficient processing techniques, avoid waste by employing manufacturing at farm gates of food crops that are seasonal and perishable. The researchers will seek joint grant application within the European Union-African Union networking initiatives.

## Data Availability

Not Applicable.

## Appendix A

**Table A1:** A list of the titles presented in the Results and Discussion section, Speakers and their backgrounds.

Title	Speaker	Background
4.1	Abiodun A. Olapade	Professor and Head of Department, Food Technology at the University of Ibadan, Ibadan, Nigeria
4.2	Frank A. Orji	Dr. Orji is Chief Research Officer, Dept. of Biotechnology, Federal Institute of Industrial Research Oshodi, Lagos, Nigeria
4.3	Sulaimon B. Kosoko	Dr. Kosoko’s special interest is in food processing and value addition. He is a Research Officer at the Food Technology Department, Federal Institute of Industrial Research Oshodi, Lagos, Nigeria. My area of intrest is food production and procees optimization to obtain a product of with optimum nutrint and functionality.
4.4	Oluwatoyin B. Oluwole	Dr. Oluwole (PhD) is the Director, Food Technology Department, Federal Institute of Industrial Research Oshodi, Lagos, Nigeria with background in Food Science and Technology. She has several years of research experience in value addition, food processing, food and nutritional health security with emphasis on functional foods, recipe manipulation for food Industries. Pilot scale production of food innovations at laboratory level is also her passion.
4.5	Michael P. Okoh	Professor at the Department of Medical Biochemistry, University of Abuja, Nigeria.
4.6	Adeola Olukosi	Dr. Olukosi is a Senior Researcher at Eko University of Medical Sciences and the Deputy Director of Research/ Head, Malaria Research Group. Department of Biochemistry and Nutrition. Nigerian Institute of Medical Research, Lagos, Nigeria.
4.7	Oyedele M. Oyeku	Dr. Dele Oyeku is the Director, Extension and Linkage at the Federal Institute of Industrial Research Oshodi, Lagos- Nigeria. He has over 25years experience in Entrepreneurship and Small Scale Industries Development; Industrial Linkage and Technology Extension Services; Sustainable Partnership and Liaison Services; Commercialization of R&D projects and Business Investment Advisory Services
4.8	Leena Favén	D.Sc. (Tech.) Leena Favén works as RDI coordinator at Centria University of Applied Sciences. She has a long experience both in international chemical industry and in publicly financed bioeconomy based RD projects. Her interests are in the development of industrial refining of high value added products from biomass based raw materials.
4.9	Mikko Junttila	Dr. Junttila is RDI specialist at Centria University of Applied Sciences with special interst in organic chemistry and biomass extraction.
4.9	Egidija Rainosalo	Dr. Rainosalo is RDI coordinator at Centria University of Applied Sciences and the Project Manager for SYMBIOMA project
4.10	Seppo Vainio	Professor, Flagship Vice-Director. Kvantum Institute, PI & Infotech Oulu, PI, Faculty of Biochemistry and Molecular Medicine, University of Oulu, Finland
4.11	Dele Raheem	Dr. Raheem is a Senior Researcher at the Arctic Centre, University of Lapland. He is also an Associate Professor in Food Microbiology, University of Helsinki, Finland.
